# Compensation Research Database: population-based injury data for surveillance, linkage and mining

**DOI:** 10.1186/s13104-016-2255-4

**Published:** 2016-10-01

**Authors:** Khic-Houy Prang, Behrooz Hassani-Mahmooei, Alex Collie

**Affiliations:** 1 Institute for Safety, Compensation and Recovery Research, Monash University, Level 18, 222 Exhibition Street, Melbourne, VIC 3000 Australia; 2 Monash University Accident Research Centre, Monash University, Clayton, VIC 3800 Australia; 3 Department of Epidemiology and Preventive Medicine, Monash University, 99 Commercial Road, Melbourne, VIC 3004 Australia

**Keywords:** Injury, Compensation, Claims, Database

## Abstract

**Background:**

Compensation health research aims to study the influence of compensation systems, processes and practices on health and health-related outcomes. In many jurisdictions, injury compensation authorities collect substantial volumes of case and service level data for the purpose of administering the compensation system. An important secondary use of such data is research and analysis to explore interactions between individuals and organisations in compensation systems, and between compensation and other systems including healthcare and legal systems, in order to understand the role of compensation processes in injury recovery.

**Results:**

The Compensation Research Database (CRD) established at the Institute for Safety Compensation and Recovery Research at Monash University, holds over 20 years of population-based data for transport and workplace injury in the state of Victoria, Australia. The CRD is unique in that it is held independently, at arm’s length from the compensation authorities that collect the data, and its primary purpose is to support research and analyses to develop new insights into system and individual level outcomes. This paper describes the core elements of the database including the design, process and type of information collected. We review some of the research findings that have been published using the CRD, and describe the ongoing program of research utilising the database.

**Conclusions:**

The CRD is a unique administrative database that supports research into compensation health, with the objective of improving understanding of the interaction between injury compensation systems and injury recovery. The availability of the CRD for independent research is leading to substantial advancements in the compensation health research field and in related areas.

## Findings

### Background

In the mid 1980s the state of Victoria, Australia established two population based, no-fault injury compensation systems. These systems provide payments for healthcare, income replacement and lifetime care costs for Victorian’s injured in transport accidents via the Transport Accident Commission (TAC) or at work via Worksafe Victoria (WSV). As of June 2015, Victoria has a population of approximately 5.9 million residents, and annually the two compensation systems accept approximately 50,000 new claims for compensation. In 2013–14, the TAC and WSV paid out over $1.1 billion and $2 billion, respectively in benefits and compensation to injured Victorians [1, 2].

In 2014, there were 249 deaths from transport accidents, 20 deaths from work accidents in the state, many thousands of serious injuries, and the burden of disease from these injuries is substantial [3–6]. Compensation systems such as those operated by the TAC and WSV play an important role in the Victorian community. As the state’s regulators and insurers of transport and workers’ compensation systems, they are responsible for compensation, funding of effective rehabilitation and accident prevention [7, 8]. They have a direct engagement with the injured person, their healthcare providers and the injured person’s employer. Furthermore, the systems in place in Victoria are similar to compensation systems throughout many other countries. The Victorian systems of injury compensation bear close resemblance to other systems in Australia, New Zealand, Canada, the United States, and Hong Kong and share some objectives (e.g., return to work after work injury) and design elements with social support systems in Europe and some South American nations [9–12].

The policies and practices of compensation systems can have a substantial impact on the health and health-related outcomes of those injured. There is growing evidence that those who receive compensation for injury or disease have poorer health and vocational outcomes and slower recovery than those with matched injuries who do not receive compensation [13, 14]. Furthermore, the magnitude of the disability is substantial, with a number of recent meta-analyses reporting moderate effect sizes for poor outcomes among those receiving compensation for their injuries than those with matched non-compensable injuries [15–18]. Conversely, a study conducted by McAllister et al. [19] in New Zealand showed that in a universal no-fault injury compensation system, those with injury who received compensation have better economic and return to work outcomes when compared with a comparative group with non-compensable impairments due to disease. Given the mixed evidence, there is a critical need for greater understanding on how the compensation system itself and individual aspects of the compensation system impact recovery following compensable injury.

One approach to attain greater understanding of the burden and outcomes of compensable injury and disease is to use compensation system data. All compensation authorities collect data for the purposes of administering, monitoring and evaluating the compensation system. In some jurisdictions, compensation data can provide detailed information on the frequency and costs of healthcare and other medical and allied health services, income replacement, and interactions with legal and other administrative systems. These data can be used to assess the impact of policies and practice changes on compensation system relevant outcomes such as cost and length of disability. These data can also be linked to healthcare datasets which enable examination of predictors of compensation system relevant outcomes. Analysis of such data will lead to a greater understanding of the role of compensation processes in injury recovery and potentially lead to improved practices and policy changes within a single jurisdiction’s healthcare and employment systems.

In 2009 and 2012, workshops led by the National Institute for Occupational Safety and Health in the United States advocated the use of workers’ compensation data to track incidence and costs, to identify priorities and gaps in workplace hazards, and to evaluate injury and illness prevention program effectiveness [20, 21]. In addition, leading organisations in the compensation health sector such as the Institute of Work and Health [22], the Partnership for Work Health and Safety in British Columbia Canada [23] and the Department of Environmental and Occupational Health Sciences at Washington University USA [24] are conducting high quality research by using workers’ compensation data to address current and emerging issues of work-related health. Overall, compensation data is a unique resource that can enable examination of population-based personal injury claims and payment records, arising from transport, workplace accidents or other compensable conditions. The aim of this paper is to describe the Compensation Research Database (CRD) and summarise the design and processes including the type of information collected, review key research findings that have been published using the CRD, and discuss research opportunities that could be examined with the CRD.

#### Compensation systems in Victoria, Australia

The state of Victoria in Australia provides no-fault compensation for both transport accidents and workplace injuries and illnesses. Those injured in land-based transport accidents involving a car, motorcycle, tram, bus or train are eligible to claim compensation for treatment, income replacement, rehabilitation and long-term support services via the TAC, regardless of fault. Individuals with mental health condition that arise subsequent to the transport accident injury are also eligible to claim compensation for mental health services. In addition, the TAC provides compensation for injury and death occurring interstate for individuals travelling in a Victorian-registered motor vehicle in other Australian states. Injuries and death occurring on the road but not involving a motorised vehicle (e.g. a collision between a pedal cyclist and a pedestrian) are not eligible for compensation. Compensation benefits cover the reasonable costs of the treatment for transport-related injuries. A medical excess is applicable ($623 for accidents between 1st July 2014 and 30th June 2015 and indexed annually according to the average weekly earnings). There are maximum fees for most services. The TAC provides funding for the following healthcare services: ambulance services (e.g. for transport from the injury location to hospital and, where required, from one hospital to another), hospital services (e.g. treatment at a public, private or rehabilitation hospital), medical services (e.g. visits to family doctor and specialist doctor), pharmacy items (e.g. for medicine prescribed by doctor and provided by a pharmacist), therapy services (e.g. physiotherapy, chiropractic, podiatry, optometry, osteopathy, and psychology) or nursing services (e.g. home visits after discharge from hospital). In addition, the TAC provides funding for income replacement and the long-term care needs of severely injured clients, including equipment for activities of daily living, modifications to housing and attendant care. Income replacement are paid in the first 18 months after the transport accident. The amount of income replacement is calculated as the weekly average of the gross earnings during the 12 months immediately before the accident date. After 18 months, if the injured person is still unable to return to work and has a severe injury, loss of earning capacity (LOEC) benefits are payable up to 3 years following the transport accident [7].

In Victoria, WSV provides compensation insurance for the majority of employers (representing approximately 85 % of the Victorian working population). WSV does not provide insurance for the proportion of the Victorian working population who are sole traders, employed at self-insuring agencies or federal government employees (approximately 15 % of the working population). All claims that exceed the financial threshold for health care expenses ($660 from 1st July 2014 and indexed annually) or requiring more than 10 days off work are required under state law to register with WSV (into effect in 1997). These cases are then managed by case managers at one of five private sector insurers contracted to WSV. To receive workers compensation benefits, workers must have their illness or injury certified by an approved medical practitioner. Workers are also eligible to lodge a claim for a mental health condition if there is a demonstrable link between work and the mental health condition. WSV provides the following compensation and services to injured workers: income replacement, medical and allied health treatments, ambulance transport, hospital treatment, personal and household help, impairment lump sums, and common law damages (where certain criteria are met). Income replacement are paid for up to 130 weeks (95 % of the pre-injury average weekly earnings (PIAWE) for the first 13 weeks and 80 % PIAWE from 14 to 130 weeks) after which point benefits cease unless the worker is considered to have a very severe and ongoing injury, usually determined via a medical assessment process [8].

### The Compensation Research Database

The Compensation Research Database (CRD) is a unique administrative database established by the third author as a platform to support research into compensation health. The CRD is held by the Institute for Safety Compensation and Recovery Research (ISCRR), an institute jointly established by the TAC, WSV and Monash University. The CRD includes data for transport and work-related claims covered by the TAC and WSV, respectively, since the mid 1980s. The data included on the two databases of interest hold over 20 years of accepted and denied population-based data of every compensable transport and workplace injury claims in the state of Victoria. Access to the CRD is made publicly available for other researchers to use, under strict guidelines approved by the compensation authorities and the Monash University Human Research Ethics Committee. Enabling data sharing of the CRD to the scientific community promotes scientific integrity, increases transparency, accelerates the impact of research by facilitating application of reusable data to new study questions, encourages and strengthens collaboration among researchers to share resources and produces new findings [25, 26].

### Data collection

The TAC and WSV have maintained administrative databases since their establishment in 1987 and 1986, respectively. These large datasets are primarily used to manage the compensation system and monitor system performance. The data is collected in accordance with the Privacy Policies of the TAC and WSV in order for the authorities to perform their statutory functions under the Accident Compensation Act 1985 [27] and Transport Accident Act 1986 [28].

Consent from TAC clients to use data for research purposes is obtained from the injured person over the telephone when a claim is lodged with the TAC (within 12 months following the date of the accident). The claim form is completed over the telephone and information regarding the transport accident are keyed directly into the TAC system. The injured person is then asked to complete a ‘General Authority to Release Information’ form which explicitly references the TAC privacy policy [29]. The privacy policy specifies that personal and health information is collected primarily for claims management and also for related purposes including accident research. The TAC also gathers information relevant to the claim from health service providers (e.g. certificate of capacity in order to access the clients’ condition and their capacity for work as a result of the injury), insurers, government agencies and employer. Following the telephone call, the TAC will make a decision to accept or deny the claim (usually within 21 days).

Similarly, consent from injured workers to use data for research purposes is obtained when a claim is lodged with the insurer who manages claims on behalf of WSV (within 30 days following the date of the accident). The injured worker completes the ‘worker’s injury claim’ form and includes a medical certificate of capacity (if claiming for weekly income payments). The worker’s injury claim form explicitly references the WSV privacy policy which provides for use of information gathered to perform the WSV functions [30]. The employer completes the ‘employer injury claim’ form. Both the worker and employer’s injury claim forms are then submitted to a WSV’s insurer by the employer. The WSV’s insurer makes preliminary assessment of the claim and enters records details on the WSV system directly. WSV also gathers information relevant to the claim from health service providers (e.g. certificate of capacity in order to access the clients’ condition and their capacity for work as a result of the injury or disease), insurers, government agencies and employer. A decision to accept or deny the claim is usually made within 28 days of claim lodgement.

**Fig. 1:**
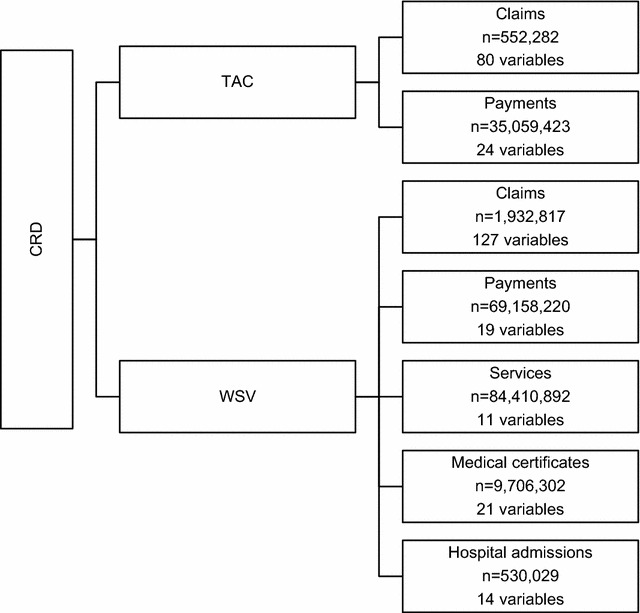
The database structure of the CRD as of December 2014

The contract establishing ISCRR as an independent research institute at Monash University funded by TAC and WSV included an agreement requiring that the TAC and WSV make de-identified administrative data available to ISCRR to support the research activities of the institute. An overview of the datasets as of December 2014 provided by the TAC and WSV are shown in Fig. 1. TAC’s claims and payments datasets are linked by a unique claim identifier. Similarly, WSV’s claims, payments, services, medical certificates and hospital admissions datasets are linked by a unique claim or payment identifier. All information received by ISCRR is potentially re-identifiable. Names and all contact details (including addresses, telephone numbers and in the case of minors, details of legal guardians) of injured individuals are removed from the data before ISCRR receives it. The data also contains details of healthcare services provided to injured individuals and the names and contact details of the service providers are also removed from the dataset prior to ISCRR receiving the data. TAC and WSV claim numbers are replaced by a ‘dummy’ identifier for the injured person. The dummy identifier is created by the organisations providing the data to ISCRR and matches each individual in the de-identified dataset to their TAC and WSV claim number. TAC and WSV maintain the key that links the dummy identifier to the claim number, and this linkage key is not available to ISCRR.

### Variables available

The information collected by the TAC, WSV or their authorised insurers includes demographics, injury, payments and treatments. Income payments are automatically recorded in the TAC and WSV systems. Information pertaining to denied claims is retained in the database. Information necessary for claims handling, from organisations such as VicRoads [31] and healthcare providers (e.g. treatment invoices), are also collected. This is all collated centrally in administrative datasets of the organisations. Tables 1 and 2 show examples of variables collected in each of the datasets. Comprehensive TAC and WSV’s data dictionaries are also available.

**Table 1:** Examples of variables collected in the TAC datasets

Group	Variables
Claimant	De-identified claim number generated by ISCRR
	Gender
	Age at the time of accident
	Date of death
	Claim status
	Role in road accident (e.g. driver, passenger, motorcyclist, pedestrian, cyclist etc.)
	Index of relative socio-economic advantage and disadvantage (IRSAD)
	Accessibility and remoteness index of Australia (ARIA)
Injury	Date of accident
	Local government area of accident
	Injury types (e.g. quadriplegia, brain injury, fractures, whiplash etc.)
	Glasgow Coma Scale (only for severe brain injury)
	Post traumatic amnesia days (only for severe brain injury)
	Total length of hospital stay
Payment	De-identified payment number generated by ISCRR
	Amount paid by the TAC for the service
	Service start date
	Benefit type code (e.g. loss of earnings, doctor, physiotherapy, psychology etc.) Item code and service description

**Table 2:** Example of variables collected in the WSV datasets

Group	Variables
Claimant	De-identified claimant number generated by ISCRR
	Gender
	Age at the time of accident
	Type of employment that the claimant is undertaking
	Occupation type major group of claimant (ANZSCO 1^st^ edition 2006)
	Index of relative socio-economic advantage and disadvantage (IRSAD)
	Accessibility and remoteness index of Australia (ARIA)
Workplace	The main group for the workplace industry codes for the activity of the claimant (ANZSIC 2006)
	The workplace industry code main group for the workplace where the injury was sustained (ANZSIC 2006)
	Workplace size
Claim	De-identified claim number generated by ISCRR
	Claim status (e.g. open, closed, or re-opened)
	First date of incapacity
Injury	Date of accident
	Mechanism and nature of injury (TOOCS 2008 3rd edition)
	First bodily location of injury major group (TOOCS 2008 3rd edition)
Payment	Payment type code (e.g. weekly compensation, doctor, physiotherapy, psychology)
	Date of invoice or the start date of a weekly payment entitlement period
	Amount listed on the invoice
Service	Service date
	First service item code
	Scheme payable amount
Medical certificate	Medical certificate types (e.g. unfit, alternative and modified duties)
	Medical examination date
	Medical certificate source (e.g. hospital, medical practitioner)

### Data quality

Data is transferred to ISCRR annually via a Secure File Transfer Protocol (SFTP). Data quality assurances are routinely conducted in-house by ISCRR staff. Rigorous examination for data completeness and accuracy using data profiling is performed on the CRD. Additional data qualities checks applied on the CRD include validation rules (e.g. service date cannot be prior to the injury date), and investigating out-of-range values (e.g. age cannot be lower than 0). Any anomalies detected in the CRD are reported back to the TAC and WSV for review.

### Data strengths and limitations

There are key advantages associated with using the CRD in research. The CRD has population coverage of transport and workplace injury in Victoria. It is readily available and therefore cost-effective to use as no data collection is required. Given the longitudinal nature of the data, the CRD also provides the opportunity to track individual over time and assess detailed service level information on a daily basis. The CRD, notably the WSV datasets use standard coding systems which are consistent with other jurisdictions in Australia. The standard coding system can also be mapped to international classification systems. More recently, the TAC has implemented the use of an international classification system such as the International Classification of Diseases, tenth edition (ICD-10). However, this is not routinely available across all claims. Finally, the CRD has the ability to be link to population surveys and population-based registries which can greatly enhance the available information about clinical or social characteristics of the population.

Despite the many advantages of using the CRD, a number of limitations must be noted. The information collected by the TAC and WSV is restricted to data required for administrative purposes. Therefore, the variables a researcher may be interested in may or may not be central to the primary record keeping. Changes to administrative procedures could change definitions and make comparison over time problematic. However, source code mapping is applied to ensure consistent coding over time. Cases of transport and workplace injury can be missed if the injured persons did not file a claim or the claim was rejected by the TAC or WSV, therefore underestimating the true prevalence. Services can be missed if the healthcare providers did not report it for billing purposes. Services not covered by the TAC or WSV and services accessed outside the compensation system are not included in the CRD. However, linking the CRD with healthcare datasets has allowed examination of services accessed outside of the compensation system. Finally, a number of claims may have missing items. Codes may also be applied incorrectly by those recording the data.

### Research utility

The CRD has been used previously to examine a number of issues including recurrent work injury [32, 33], work absences and disability [34, 35], return to work [36–39], age and gender differences [40, 41], incidence of certain work-related injury such as occupational dermatitis [42] and brain injury [43], risk factors for specific occupations groups such as ambulance and paramedics officers [44], and healthcare service use [45–50]. In addition, the CRD has been linked to numerous state and national datasets [51–53]. Some of these issues are described below.

#### Return to work

One area of research where the CRD has been applied is return to work. Return to work is an important step in recovering from an injury, returning to a normal life and reducing the financial and emotional burden on the individual and their family. The CRD has been used to identify predictors of sustained return to work following a work-related injury or disease [36] and to assess the impact of an aging workforce on return to work [37]. In addition, the CRD has been used to explore the role of the general practitioner (GP) in facilitating return to work [38, 39]. The CRD has enable identification of patterns and trends of GP certification behaviour by exploring the types and duration of medical certificates issued by a GP on various injury types. Identifying various individual, organisational and healthcare factors that influence return to work has allowed identification of ‘high-risk’ groups for return to work interventions, redesign of capacity medical certificates by WSV, and development of new guidelines for medical certification by a GP.

#### Healthcare service use

The CRD can identify all individuals seen by a particular healthcare service provider due to the information provided as part of their treatment and billing practices. This enables the CRD to produce a profile of all services received by each individual and their associated costs over any defined time period. The CRD has been previously used to explore healthcare service utilisation following work-related musculoskeletal disorders [45], transport-related injuries [46, 47] including whiplash injury [48] and traumatic brain injury [49, 50]. The CRD allows detailed examination of the use and costs of healthcare services which are important for the planning of resources and developing public policies.

#### Data linkages

Aside from investigation of issues unique to each scheme, the CRD provides a platform for linking data to other datasets that enable large-scale epidemiological studies. The CRD is a potentially re-identifiable dataset. It cannot be directly linked to external datasets using common identifiers such as name and date of birth as these are de-identified. However the TAC and WSV maintain a linkage key that enables data linkage with their active involvement and approval [51]. In this way, it is possible to link external datasets to the CRD on a case-by-case basis, whilst maintaining the de-identified nature of the database for routine analysis and surveillance activity.

The CRD has been linked to injury registries already managed by Monash University including the Victorian Orthopaedic Trauma Outcomes Registry (VOTOR). VOTOR monitors and evaluates the care of orthopaedic injured patients in Victoria. Linking the CRD with VOTOR has helped to improve knowledge of the drivers of claim costs, claim durations and the relationship with patient outcomes [52]. The CRD has been linked with Medicare, the Australian universal healthcare system. Medicare provides all Australians with free or low-cost access to medical and hospital care. It is financed by the Australian government, with funding from income taxes and the Medicare levy. The Medicare levy is a surcharge for individuals with income above a certain level who do not have private health insurance [54]. Linking the CRD with Medicare has enabled detailed examination of pre-existing health conditions, medications and health service use and how these impact transport injury rates and transport injury outcomes [51, 53]. The CRD has recently been linked to hospital datasets including the Victorian Admitted Episodes Dataset (VAED) and the Victorian Emergency Minimum Dataset (VEMD) via the Victorian Department of Human Services and Health. Linking the CRD with the VAED and VEMD will allow thorough examination of prior health service utilisation on recovery from work-related injuries. The CRD provides a unique platform that leverages existing data and linkages in order to evaluate the impacts of transport and workers’ compensation schemes on outcomes for injured Victorians.

### Future activities

More recently ISCRR has developed an enhanced program of data activities that expands on the existing work to date to deliver new insights and innovations to TAC and WSV. The program will contain three key streams of work. The first stream will be the expansion of the existing CRD activities including data management, the second stream will involve linking and comparing compensation data to external sources of information of relevance to the compensation system (data linkage as described above), and the third stream is designed to use innovative methods for predictive mining and analysing data to deliver new insights. These future planned CRD activities, with the exclusion of data linkages are described below. An example of how the CRD can be applied to a ‘natural experiment’ is also provided below.

#### Data management

Additional available data sources from the TAC and WSV will be incorporated into the CRD for research use. This includes medical certificates issued for TAC clients, and workplace safety inspections reports undertaken by WSV. Furthermore, existing dataset such as the WorkHealth program conducted by WSV [55] and newly developed datasets such as the Victorian Working Population Survey will be added to ISCRR’s data holdings. These datasets will be housed and managed by ISCRR and made available for research use using the protocols established for the CRD. WorkHealth is a program which aims to promote the benefits of a healthier workforce, to reduce Victorian workers’ risk of chronic preventable diseases such as type 2 diabetes and cardiovascular diseases and to explore the links between chronic disease and workplace injury. One part of the program involved Victorian workers completing a 15 min voluntary health risk assessment. To date, 800,000 health checks records of Victorian workers are available in the WorkHealth dataset. The Victorian Working Population Survey is currently under development. The project will annually survey eligible Victorian workforce (employed and unemployed persons) to provide a monitor of perceptions relating to work-related physical and mental health risks and hazards. In addition to hazard perception, the survey will monitor attitudes to health and safety and the recollection and perception of incidents among individuals as well as perceptions of ‘reasonable’ risk, mental and physical health status, job satisfaction, work hours, industry, appointment description, intention to leave, duration of employment, education, and other related labour-related and demographic details.

#### Data analytics

Data analytics is designed to use innovative methods for predictive mining [56] and analysing data to deliver new insights, with a strong focus on risk prediction, and emerging economic, social and demographic issues of concern. Advanced predictive mining and forecasting techniques will be applied to the CRD in order to answer questions that cannot be addressed using conventional statistical techniques. For example, how the combination of order, frequency, the gap between, and the delay in providing medical and paramedical services may lead to different mental health recovery outcomes. In addition, the large number of claims and the availability of service data at the daily level enable the researchers to study claimant profiles and recovery trend clusters. The CRD is also expected to be embedded in a dynamic complex model such as agent-based computational models of claims management, and be used for high resolution visualization models [57, 58]. The high resolution visualization models will allow effective communication of findings and key messages to the compensation health community, decision-makers and the public. Furthermore, the availability of unstructured data such as medical reports held by the compensation authorities will allow application of text analytics to areas not yet examined. Emerging economic, social and demographic issues of concern in Victoria can also be investigated using the CRD. For example, analyses can be conducted to understand the dynamics of recovery and compensation under macro-level changes such as an aging workforce, aging drivers’ population, economic cycles, decreasing share of employment in manufacturing, and changing gender composition in the workplace.

#### Example of CRD application as a natural experiment

One of the key future applications of the CRD will be in designing and studying ‘natural experiments’. A natural experiment usually takes the form of an observational study in which the researcher cannot control or withhold the allocation of an intervention to particular areas or communities, but where natural or pre-determined variation in allocation occurs [59]. An example of a natural experiment can be examining the social and economic responses to a financial crisis [60, 61]. To conclude this section, we briefly present some evidence on how the CRD can be applied to study the impact of the recent global financial crisis (GFC) on work-related injury claims in Victoria, using trend analyses and forecasting. According to the literature [62, 63], the impact of the GFC on the number and duration of work-related injury compensation claims has been mixed. In contrast, the available evidence in Australia suggested that fewer claims were lodged during the time of crisis [62].

**Fig. 2:**
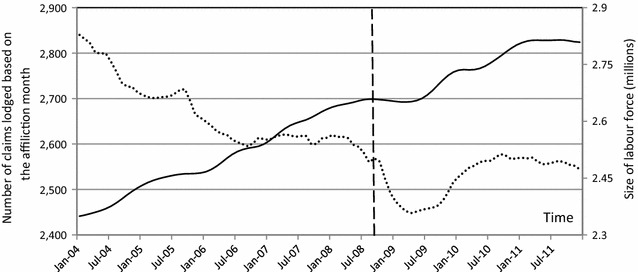
Size of Victorian labour force (*solid line*) and number of work-related injury claims (*dotted line*) in Victoria. September 2008 is marked by a *dashed line*

Figure 2 presents two trends. The solid line shows the total number of workers employed in Victoria between January 2004 and December 2011 [64] and the dotted line shows the trend^1^ of standard^2^ claims for injured workers aged 15–65 years in Victoria over the same time period. The number of claims has significantly decreased from October 2008 (the start of the GFC in Australia), reaching its lowest level by April 2009, and then recovering over the next 12 months. A potential reason behind this pattern is the high level of unemployment during the GFC, which led to a decline of workers in the workforce and in turn resulted in fewer injuries. However, the downward trend observed in the Victorian labour force (solid line) does not appear to be as substantial as the downward trend observed in claims lodgement (dotted line).

**Fig. 3:**
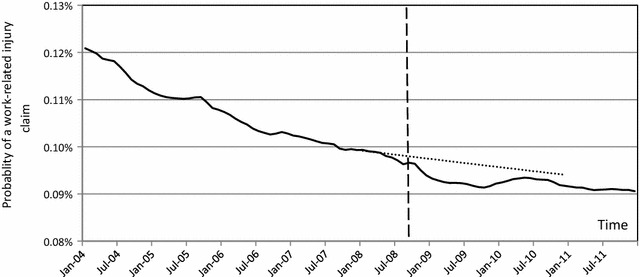
Probability of a work-related injury claim being lodged per worker (*solid line*). The *dotted line* uses the data until January 2008 to forecast the next 3 years. September 2008 is marked by a *dashed line*

Figure 3 presents the probability of a work-related injury claim per worker in Victoria by month of injury^3^ (solid line). To highlight the impact of the GFC, the data between January 2004 and December 2007 is used to forecast the trend for the period of January 2008 and December 2010 (dotted line in Fig. 3). As the figure shows, after controlling for the size of labour force in Victoria, it appears that the GFC was associated with fewer work-related injury claims lodged for compensation. Guthrie et al. [62] suggested that a decrease in claims lodgement may be due to the slower pace of work and workers’ fear of job loss. Using the available data in the CRD, this analysis can be broadened to study the impact of the GFC on the dynamics of work-related injury claims across age groups, gender, industries, injury types, income levels and workplace sizes.

## Availability and requirements

Researchers including those based outside of Australia who are interested in accessing the CRD can access data on request. Researchers must be able to demonstrate that the data will be securely stored and protected. Information related to the process for requesting data is contained on ISCRR’s website at: http://www.iscrr.com.au. Prior to submitting a data request, a meeting with an ISCRR representative from the CRD data team is necessary to understand the researcher’s data needs, provide an overview of the data within the CRD and discuss any data-related issues pertaining to the data request. The provision of the data extract may be subject to a fee-for-service, and will be discussed with the researcher in advance of submitting their data request. The CRD is governed by a steering committee including representative of ISCRR, Monash University, the TAC and WSV. Steering committee meetings are held quarterly or more often if required. Access to the data is subject to the conditions of the CRD data access policy and approval of the steering committee. The following criteria (but not limited to) are taken into consideration when reviewing the data request; (a) demonstrated reasonable need for the requested data to answer the specified research question(s), (b) the use of data is not harmful to the individuals that the information is about, (c) the proposed research is clearly in the public interest, (d) the proposed research is consistent with ISCRR’s research strategy, (e) scientific merit (peer- or merit-reviewed), (f) ethical considerations. Once the data request is approved by the CRD steering committee, a de-identified password protected dataset (i.e. CVC, SAS, SPSS data file) will be made available to the researcher via SFTP. Researchers are required to submit all research outputs for review to the CRD steering committee prior to public disclosure.

In Australian compensation health research, the process and governance approach to the CRD is unique. Presently, in many jurisdictions in Australia, researchers are unable to access compensation data freely. Access is provided on a specific request basis to a compensation authority in some other jurisdictions internationally. In contrast, the CRD is held independently and at arm’s length from the compensation authority via a third party entity. Given the growing field of research on open data [25, 26], opening access and sharing of the CRD worldwide to researchers enables scientific integrity, increases transparency, participation and collaboration.

## Conclusions

The CRD was designed to investigate the role of compensation processes in injury recovery and to identify emerging areas in compensation health. Given the limited availability of data available in this growing research field, researchers who are interested in compensation health would find the CRD rich in information and invaluable in their future studies.

## Abbreviations

CRD: Compensation Research Database
ISCRR: Institute for Safety, Compensation and Recovery Research
GP: General Practitioner
GFC: global financial crisis
SFTP: secure file transfer protocol
TAC: Transport Accident Commission
VAED: Victorian admitted episodes dataset
VEMD: Victorian emergency minimum dataset
VOTOR: Victorian orthopaedic trauma outcomes registry
WSV: Worksafe Victoria

## Footnotes

1 Trend is measured after removing the seasonal and random components of the original claims data using time series decomposition.

2 A standard claim is a claim for time loss/weekly compensation which exceeds the employers’ excess ($660 from 1st July 2014 and indexed annually) or requiring more than 10 days off work.

3 It is calculated by dividing the number of claims (dashed line in Fig. 2) by the number of workers (solid line in Fig. 2).

## References

[1] Transport Accident Commission. Annual report. 2014.

[2] Work Cover Authority. Annual report. 2014.

[3] SafeWork Australia (2014). Work-related traumatic injury fatalities, Australia 2013.

[4] Transport Accident Commission. Road toll—annual. 2014 [9 Feb 2015]. http://www.tac.vic.gov.au/road-safety/statistics/road-toll-annual.

[5] Victoria WorkSafe (2012). WorkSafe Victoria statistical summary 2011/12.

[6] Transport Accident Commission (2014). Road safety statistical summary.

[7] Transport Accident Commission. https://www.tac.vic.gov.au/. Accessed 30 Jun 2015.

[8] WorkSafe Victoria. http://www.worksafe.vic.gov.au/. Accessed 30 Jun 2015.

[9] SafeWork Australia. Comparison of workers’ compensation arrangements in Australia and New Zealand, 2012–2013. Canberra, ACT, 2014.

[10] LaDou J (2011). The European influence on workers’ compensation reform in the United States. Environ Health..

[11] Walters D. An international comparison of occupational disease and injury compensation schemes. A research report prepared for the Industrial Injuries Advisory Council (IIAC). Cardiff Work Environment Research Centre, Cardiff University, 2007.

[12] Dembe AE (2010). How historical factors have affected the application of workers’ compensation data to public health. J Public Health Pol..

[13] Gabbe B, Cameron P, Williamson O, Edwards E, Graves S, Richardson M (2007). The relationship between compensable status and long-term patient outcomes following orthopaedic trauma. Med J Aust.

[14] Cameron I, Rebbeck T, Sindhusake D, Rubin G, Feyer A, Walsh J (2008). Legislative change is associated with improved health status in people with whiplash. Spine..

[15] Harris I, Mulford J, Solomon M, van Gelder JM, Young J (2005). Association between compensation status and outcome after surgery: a meta-analysis. JAMA.

[16] Elbers NA, Hulst L, Cuijpers P, Akkermans AJ, Bruinvels DJ (2013). Do compensation processes impair mental health? A meta-analysis. Injury..

[17] Binder LM, Rohling ML (1996). Money matters: a meta-analytic review of the effects of financial incentives on recovery after closed-head injury. Am J Psychiatr.

[18] Murgatroyd DF, Casey PP, Cameron ID, Harris IA (2015). The effect of financial compensation on health outcomes following musculoskeletal injury: systematic review. PLoS ONE.

[19] McAllister S, Derrett S, Audas R, Herbison P, Paul C (2013). Do different types of financial support after illness or injury affect socio-economic outcomes? A natural experiment in New Zealand. Soc Sci Med.

[20] Utterback DF, Schnorr TM. Use of workers’ compensation data for occupational safety and health: Proceedings from June 2012 Workshop. National Institute for Occupational Safety and Health, 2013.

[21] Utterback DF, Schnorr TM. Use of workers’ compensation data for occupational injury & illness prevention. National Institute for Occupational Safety and Health, 2010.

[22] Institute for Work and Health. http://www.iwh.on.ca. Accessed 30 Jun 2015.

[23] Partnership for work health and safety. http://pwhs.ubc.ca/. Accessed 30 Jun 2015.

[24] University of Washington: Department of environmental and occupational health sciences. http://deohs.washington.edu/occepi. Accessed 30 Jun 2015.

[25] Institute of Medicine (US). The benefits of data sharing. Sharing clinical research data: Workshop summary. Washington (DC): National Academic Press (US); 2013. 23700647

[26] Piwowar HA, Becich MJ, Bilofsky H, Crowley RS (2008). On behalf of the caBIG data sharing and intellectual capital warehouse. towards a data sharing culture: recommendations for leadership from academic health centers. PLoS Med..

[27] Accident compensation act 1985. http://www.austlii.edu.au/au/legis/vic/consol_act/aca1985204/. Accessed 9 Feb 2015.

[28] Transport accident act 1986. http://www.austlii.edu.au/au/legis/vic/consol_act/taa1986204/. Accessed 9 Feb 2015.

[29] Transport Accident Commission. Privacy Policy. http://www.tac.vic.gov.au/claims/what-the-tac-pays-for/policy/clients-turning-18-years-of-age/tac-privacy-policy/TAC-Privacy-Policy.pdf. Accessed 9 Feb 2015.

[30] WorkSafe Victoria. Privacy Policy. 2013. http://www.worksafe.vic.gov.au/__data/assets/pdf_file/0004/35815/WSV1595-Privacy-Policy_web.pdf. Accessed 9 Feb 2015.

[31] VicRoads. https://www.vicroads.vic.gov.au/. Accessed 30 Jun 2015.

[32] Ruseckaite R, Collie A (2013). The incidence and impact of recurrent workplace injury and disease: a cohort study of WorkSafe Victoria, Australia compensation claims. BMJ Open..

[33] Ruseckaite R, Clay FJ, Collie A (2012). Second workers’ compensation claims: who is at risk? Analysis of WorkSafe Victoria, Australia compensation claims. Can J Public Health.

[34] Berecki-Gisolf J, Collie A, McClure R (2013). Work disability after road traffic injury in a mixed population with and without hospitalisation. Accid Anal Prev.

[35] Smith P, Black O, Keegel T, Collie A (2014). Are the predictors of work absence following a work-related injury similar for musculoskeletal and mental health claims?. J Occup Rehabil.

[36] Berecki-Gisolf J, Clay F, Collie A, McClure R (2012). Predictors of sustained return to work after work-related injury or disease: insights from workers’ compensation claims records. J Occup Rehabil.

[37] Berecki-Gisolf J, Clay FJ, Collie A, McClure RJ (2012). The impact of aging on work disability and return to work: insights from workers’ compensation claim records. J Occup Environ Med.

[38] Collie A, Ruseckaite R, Brijnath B, Kosny AA, Mazza D (2013). Sickness certification of workers compensation claimants by general practitioners in Victoria, 2003–2010. Med J Aust.

[39] Ruseckaite R, Collie A, Bohensky M, Brijnath B, Kosny A, Mazza D (2014). Trends in sickness certification of injured workers by general practitioners in Victoria, Australia. J Occup Rehabil.

[40] Berecki-Gisolf J, Smith PM, Collie A, McClure RJ (2015). Gender differences in occupational injury incidence. Am J Ind Med.

[41] Smith PM, Berecki-Gisolf J. Age, occupational demands and the risk of serious work injury. Occup Med. 2014. 10.1093/occmed/kqu125 25168227

[42] Lyons G, Keegel T, Palmer A, Nixon R (2013). Occupational dermatitis in hairdressers: do they claim workers’ compensation?. Contact Dermat.

[43] Chang VC, Ruseckaite R, Collie A, Colantonio A. Examining the epidemiology of work-related traumatic brain injury through a sex/gender lens: analysis of workers’ compensation claims in Victoria, Australia. Occupational and Environmental Medicine. 2014. 10.1136/oemed-2014-102097 25052083

[44] Roberts MH, Sim MR, Black O, Smith P. Occupational injury risk among ambulance officers and paramedics compared with other healthcare workers in Victoria, Australia: analysis of workers’ compensation claims from 2003 to 2012. Occupational and Environmental Medicine. 2015. 10.1136/oemed-2014-102574 25780033

[45] Berecki-Gisolf J, Collie A, McClure R (2013). Determinants of physical therapy use by compensated workers with musculoskeletal disorders. J Occup Rehabil.

[46] Ruseckaite R, Gabbe B, Vogel AP, Collie A (2012). Health care utilisation following hospitalisation for transport-related injury. Injury..

[47] Elbers NA, Cuijpers P, Akkermans AJ, Collie A, Ruseckaite R, Bruinvels DJ (2013). Do claim factors predict health care utilization after transport accidents?. Accid Anal Prev.

[48] Berecki-Gisolf J, Collie A, McClure RJ (2013). Reduction in health service use for whiplash injury after motor vehicle accidents in 2000–2009. Results from a defined population. J Rehabil Med.

[49] Prang K-H, Ruseckaite R, Collie A (2012). Healthcare and disability service utilization in the 5-year period following transport-related traumatic brain injury. Brain Inj.

[50] Collie A, Prang KH (2013). Patterns of healthcare service utilisation following severe traumatic brain injury: an idiographic analysis of injury compensation claims data. Injury..

[51] Berecki-Gisolf J, Collie A, Hassani-Mahmooei B, McClure R (2015). Use of antidepressant medication after road traffic injury. Injury..

[52] Gabbe B, Simpson P. Victorian orthopaedic trauma outcomes registry. 2012. http://www.iscrr.com.au/reports-pubs/research-reports/votor-client-outcomes.pdf.

[53] Hassani-Mahmooei B, Berecki-Gisolf J, Hahn Y, McClure R. The cost of comorbidity to the transport accident commission compensation scheme. 2014. http://www.iscrr.com.au/reports-pubs/research-reports/comorbidty_executive_summary.pdf.

[54] Australian Government: department of human services. Medicare: looking after the health of Australians. http://www.humanservices.gov.au/customer/dhs/medicare. Accessed 30 Jun 2015.

[55] WorkSafe Victoria. About WorkHealth. http://www.worksafe.vic.gov.au/safety-and-prevention/health-and-wellbeing/about-workhealth. Accessed 30 Jun 2015.

[56] Bellazzi R, Zupan B (2008). Predictive data mining in clinical medicine: current issues and guidelines. Int J Med Inform.

[57] Martinez R, Ordunez P, Soliz PN, Ballesteros MF. Data visualisation in surveillance for injury prevention and control: conceptual bases and case studies. Inj Prev. 2016. 10.1136/injuryprev-2015-041812 PMC4833094 26728006

[58] Shneiderman B, Plaisant C, Hesse BW. Improving health and healthcare with interactive visualization methods. HCIL Technical Report. 2013.

[59] Petticrew M, Cummins S, Ferrell C, Findlay A, Higgins C, Hoy C (2005). Natural experiments: an underused tool for public health?. Publ Health..

[60] Hodson D, Quaglia L (2009). European perspectives on the global financial crisis: introduction. J Common Mark Stud.

[61] Nabin M, Bhattacharya S, Rafiq S (2015). Mortgage-Backed Securities (MBS): is it a curse or a blessing for the Australian home loan market? A natural experiment. Aust Econ Pap.

[62] Guthrie R, Aurbach R, Fronsko A (2010). Workers’ compensation and economic downturn: predictions and reflections. J Soc Secur Work Compens.

[63] Hartwig RP (1997). Riding the economic cycles: how growth and recession affect workers’ compensation. Compens Benefits Rev.

[64] Australian bureau of statistics. Labour force, Australia. 2015. http://www.abs.gov.au/AUSSTATS/abs@.nsf/Lookup/6202.0Main+Features1Apr%202015?OpenDocument. Accessed 30 Jun 2015.

